# *Desulfuribacillus alkaliarsenatis* gen. nov. sp. nov., a deep-lineage, obligately anaerobic, dissimilatory sulfur and arsenate-reducing, haloalkaliphilic representative of the order *Bacillales* from soda lakes

**DOI:** 10.1007/s00792-012-0459-7

**Published:** 2012-05-24

**Authors:** D. Y. Sorokin, T. P. Tourova, M. V. Sukhacheva, G. Muyzer

**Affiliations:** 1 Winogradsky Institute of Microbiology, Russian Academy of Sciences, Prospect 60-let Octyabrya 7/2, 117811 Moscow, Russia; 2 Environmental Biotechnology Group, Department of Biotechnology, Delft University of Technology, Delft, The Netherlands; 3 Centre “Bioengineering”, Russian Academy of Sciences, Prospect 60-let Octyabrya 7/1, 117312 Moscow, Russia; 4 Department of Aquatic Microbiology, Institute for Biodiversity and Ecosystem Dynamics, University of Amsterdam, Amsterdam, The Netherlands

**Keywords:** Soda lakes, Sulfur-reducing, Arsenate-reducing, Haloalkaliphilic

## Abstract

An anaerobic enrichment culture inoculated with a sample of sediments from soda lakes of the Kulunda Steppe with elemental sulfur as electron acceptor and formate as electron donor at pH 10 and moderate salinity inoculated with sediments from soda lakes in Kulunda Steppe (Altai, Russia) resulted in the domination of a Gram-positive, spore-forming bacterium strain AHT28. The isolate is an obligate anaerobe capable of respiratory growth using elemental sulfur, thiosulfate (incomplete reduction) and arsenate as electron acceptor with H_2_, formate, pyruvate and lactate as electron donor. Growth was possible within a pH range from 9 to 10.5 (optimum at pH 10) and a salt concentration at pH 10 from 0.2 to 2 M total Na^+^ (optimum at 0.6 M). According to the phylogenetic analysis, strain AHT28 represents a deep independent lineage within the order *Bacillales* with a maximum of 90 % 16S rRNA gene similarity to its closest cultured representatives. On the basis of its distinct phenotype and phylogeny, the novel haloalkaliphilic anaerobe is suggested as a new genus and species, *Desulfuribacillus alkaliarsenatis* (type strain AHT28^T^ = DSM24608^T^ = UNIQEM U855^T^).

**Electronic supplementary material:**

The online version of this article (doi:10.1007/s00792-012-0459-7) contains supplementary material, which is available to authorized users.

## Introduction

Our recent study on sulfidogenesis in anoxic sediments of hypersaline soda lakes in central Asia demonstrated a high activity of sulfur respiration even at soda-saturating conditions, in contrast to sulfate reduction (Sorokin et al. 2010). Our explanation for this is that at high pH the real substrate for sulfur respiration is linear soluble polysulfides S_x_^2−^ (Hedderich et al. 1999) which are formed due to a fast abiotic reaction between sulfide and elemental sulfur, and which are chemically stable at alkaline conditions. The use of polysulfides as substrate makes the whole process energetically much more favorable, because of the absence of the energy expensive sulfate activation step. Furthermore, sulfur respiration is energetically favored at high pH since protons are released.

So far, very little is known on the identity of sulfur-respiring prokaryotes functioning at high pH and high salt. Up to now, only two highly specialized haloalkaliphilic sulfur-reducing bacteria have been isolated from soda lakes. Moderately salt-tolerant isolates utilizing fatty acids as electron donor have been described as a novel genus and species *Desulfurispira natronophila* (Sorokin and Muyzer 2010), while an extremely natronophilic organism growing with H_2_, formate and acetate is a member of the order *Halanaerobiales* and described as a novel species *Natroniella sulfidigena* (Sorokin et al. 2011).

In this report, we describe the properties of a novel obligately anaerobic haloalkaliphilic bacterium isolated from Siberian soda lakes with a broader dissimilatory metabolism, which, apart from elemental sulfur, can also grow by arsenate reduction.

## Methods

### Samples

Sediment samples of the top 10 cm layer from six soda lakes in southern Kulunda Steppe (Altai, Russia, July 2009) were mixed in equal proportions to make a single inoculum used to enrich for sulfur-respiring haloalkaliphiles. The soda lakes had a pH range of 10.05–10.85, a salinity from 70 to 400 g l^−1^, a soluble carbonate alkalinity from 0.8 to 4.7 M, and the free sulfide content of the sediments from 0.28 to 3.17 mM (Supplementary Table S1).

### Enrichment and cultivation

Enrichment and routine cultivation of haloalkaliphilic anaerobes were performed at 28 °C on a mineral medium containing sodium carbonate buffer (0.5 M Na^+^) with pH 10, 0.1 M NaCl, and 0.5 g l^−1^ of K_2_HPO_4_. After sterilization in closed bottles, the medium was supplemented with 50 mM formate as carbon and energy source, 1 mM acetate as carbon source, 10 mg l^−1^ of yeast extract, 4 mM NH_4_Cl, 1 mM MgSO_4_, 1 ml l^−1^ each of an acidic trace metal solution and vitamin mix (Pfennig and Lippert 1966), and 1 ml l^−1^ of an alkaline Se/W solution (Plugge 2005). Elemental sulfur (Fluka) was autoclaved as a thick water paste at 110 °C for 40 min in closed bottles and added in excess of approximately 3 g l^−1^. Other electron acceptors used were KNO_3_ and Na_2_S_2_O_3_ (20 mM each), KNO_2_, Na_2_SO_3_, sodium selenate and selenite, sodium arsenate, DMSO (5 mM each), sodium fumarate (20 mM), and freshly prepared amorphous ferrihydrite (20 mM). The medium was dispensed into either Hungate tubes or serum bottles capped with butyl rubber stoppers and made anoxic by 5 cycles of flushing with argon gas-evacuation. In all cases, except for selenate, selenite and ferrihydrite, 1 mM HS^−^ was added to the medium as a reductant. Growth at micro-oxic conditions was tested with an oxygen concentration in the gas phase of 2 %. Routine cultivation was performed either in 15 ml Hungate tubes with 10 ml medium (soluble electron acceptors), or in 50 ml serum bottles with 40 ml medium with argon in the gas phase in case of sulfur and ferrihydrite. The pH dependence was examined at Na^+^ content of 0.6 M, using the following filter-sterilized buffer systems: for pH 6–8, 0.1 M HEPES and NaCl/NaHCO_3_; for pH 8.5–11, a mixture of sodium bicarbonate/sodium carbonate containing 0.1 M NaCl. To study the influence of salt concentration on growth, sodium carbonate buffers at pH 10 containing 0.1 and 3 M of total Na^+^ were mixed in different proportions.

### Analytical procedures

Free sulfide and the sulfane atom of polysulfide were measured colorimetrically (Trüper and Schlegel 1964) after precipitation in 10 % (w/v) Zn-acetate. The internal sulfur of the polysulfide was separated by acidification of the sample to pH < 3 with concentrated HCl, precipitated by centrifugation, washed with distilled water, dried, extracted from the cell pellet with acetone and determined by cyanolysis (Sörbo 1957). Cell protein was determined according to Lowry et al. (1951) after removal of sulfide/polysulfide and washing the cell pellet several times with 0.6 M NaCl. Arsenite was detected by anionic chromatography after neutralization of the supernatant using Biotronic IC-1000 chromatograph (Germany), anion-exchange column BT11AN, conductometer detector and 1 mM Na_2_CO_3_/1.2 mM NaHCO_3_ as eluent with a flow rate of 1.5 ml min^−1^. Acetate was analyzed in the filtered supernatant after acidification to pH 4 by anionic chromatography (Biotronic IC-1000; column BT III OS; conductivity detector; 1 mM HCl as eluent, 0.8 ml min^−1^). Phase-contrast microphotographs were obtained with a Zeiss Axioplan Imaging 2 microscope (Göttingen, Germany). For electron microscopy, the cells were negatively contrasted with 1 % (w/v) neutralized phosphotungstate and observed in JEOL 100 (Japan) transmission electron microscope. Polar lipids were extracted from 50 mg of freeze-dried cells with acidic methanol and the fatty acid methyl esters were analyzed by GC–MS according to Zhilina et al. (1997). The cell wall fractionation and its peptidoglycan analysis were performed according to Streshinskaya et al. (1979). To analyze the respiratory quinones, the cells were extracted with cold acetone and the eluate was subjected to TLC (Collins 1985). A major UV-absorbing band was eluted and subjected to tandem mass spectrometry (LCG Advantage Max) with chemical ionization at atmospheric pressure and the quinones were identified by ionic mass.

### Genetic and phylogenetic analysis

The isolation of the DNA and determination of the G + C content of the DNA was performed according to Marmur (1961) and Marmur and Doty (1962), respectively. For molecular analysis, the DNA was extracted from the cells using lysis in 1 % (w/v) SDS/0.2 M NaOH at 60 °C and purified with the Wizard Preps Kit (Promega, USA). The nearly complete 16S rRNA gene was obtained using general bacterial PCR primers 11f and 1492r (Lane 1991). The preliminary analysis of the new sequences was done with NCBI BLAST server (http://www.ncbi.nlm.nih.gov/BLAST/) and RDP CLASSIFIER server (http://rdp.cme.msu.edu/classifier/classifier.jsp). The *aar*A gene (respiratory arsenate reductase subunit A) amplification was carried out with two primer sets: for a short fragment the pair aarAf/aarAr (Malasarn et al. 2004) and for a longer fragment—with the HAArrA-D1F/HAArrA-D1R pair (Kulp et al. 2006). The sequences were aligned with sequences from the GenBank using CLUSTAL W and the phylogenetic tree was reconstructed using neighbor-joining algorithm in the TREECONW program package (van de Peer and de Wachter 1994) and maximum-likelihood algorithm in PHYLIP 3.5c software (Felsenstein 1993).

## Results and discussion

### Enrichment and isolation of pure cultures

**Fig. 1:**
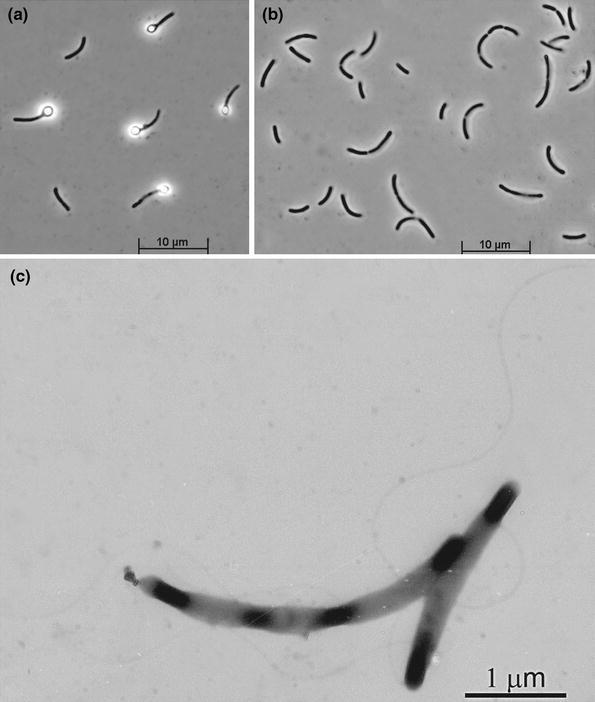
Cell morphology of strain AHT28 grown at pH 10 either with sulfur (**a**) or arsenate (**b**, **c**) as electron acceptor. **a**, **b** Phase-contrast microphotographs, **c** electron microphotograph of positively stained cells

An anaerobic enrichment from soda lake sediments with formate as electron donor and sulfur as electron acceptor at 0.6 M total Na^+^ and pH 10 resulted in a rapid formation of yellow polysulfide with total sulfane concentration reaching 12 mM within 1 week. No growth was observed in control incubations lacking either formate or sulfur. The positive culture was dominated by slender, motile bend rods forming round terminal endospore (Fig. 1). The dominant phenotype was isolated in pure culture in a single round of serial dilutions and designated strain AHT28.

### Identification

**Fig. 2:**
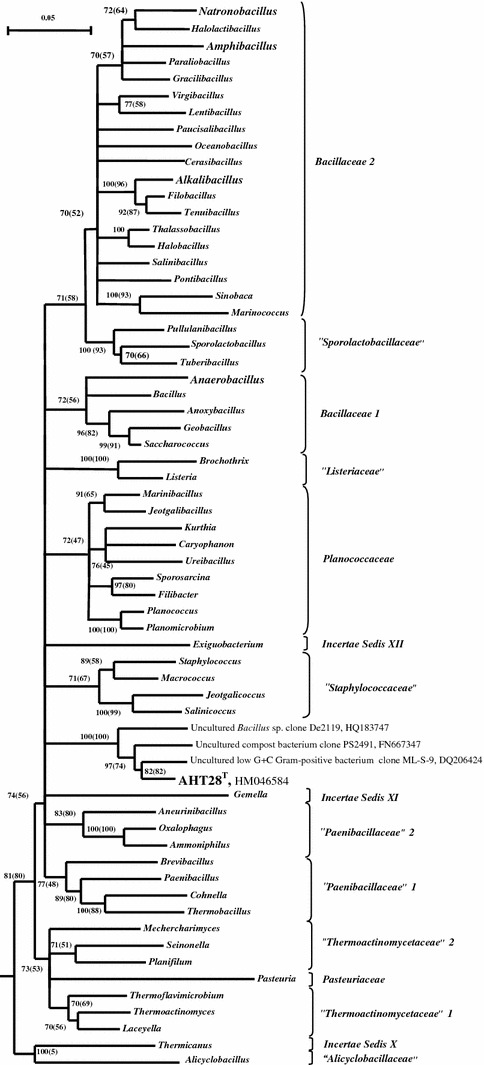
Phylogenetic position of the strain AHT28 within the order *Bacillales* based on 16S rRNA gene sequence analysis. The phylogenetic outline of the order is according to Ludwig et al. (2009), where each branch represents a consensus from the sequences of several species in each genus. Tree topography and evolutionary distances are obtained by the neighbor-joining method with Jukes and Cantor distances. *Numbers at the nodes* indicate the percentage of bootstrap values in 1000 replications (*numbers in parenthesis* indicate bootstrap values obtained by the alternative maximum-likelihood algorithm). Only values above 70 % are included. *Multifurcations* indicate that a common relative branching order was not significantly supported by the bootstrap analysis

Phylogenetic analysis placed strain AHT28 into the order *Bacillales* as a deep independent lineage with a maximum of 90 % 16S rRNA gene sequence similarity to its nearest described species but without any obvious association with either of the families (Fig. 2). The closest sequences present in the GenBank belongs to an uncultured low GC Gram-positive bacterium, clone ML-S-9 (96 % similarity), obtained from haloalkaline Mono Lake, in California, USA (Hollibaugh et al. 2006) and to two other uncultured bacteria (93–94 % similarity) from different habitats. The RDP database query indicated 100 % probability that the sequence belongs to the order *Bacillales* but did not place it in any known families of the order. Most probably, the strain and its close uncultured relatives represent a novel family but in our opinion more isolates are necessary to obtain to make a firm phenotypic assessment of this novel group of anaerobic bacilli.

The analysis of cellular fatty acids in polar lipids of strain AHT28 showed a lack of C15 components diagnostic for the low G + C *Firmicutes* (Kämpfer 2002), and a domination, instead, of C16 fatty acids. The same major components were detected in anaerobic alkaliphile *Anaerobacillus alkalilacustris* (Zavarzina et al. 2009) obtained from a Siberian soda lake. In addition, strain AHT28 contained high concentrations of unsaturated C18 (Supplementary Table S2). Peptidoglycan analysis of the cell wall demonstrated a presence of meso-DAP, a typical marker of the order *Bacillales* (Schleifer and Kandler 1972), and the quinone analysis identified MK-7 as the major respiratory lipoquinone.

### Physiology

**Fig. 3:**
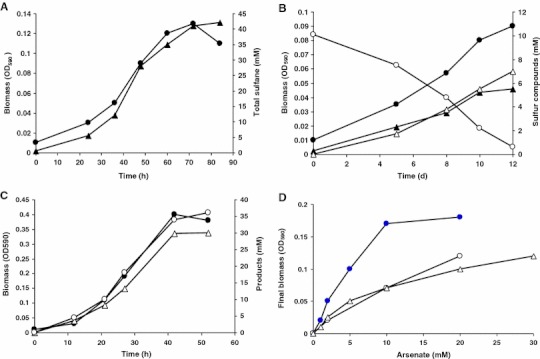
Anaerobic growth dynamics of strain AHT28 at pH 10 and 0.6 M total Na^+^. **a** Growth with 50 mM formate/2 mM acetate and elemental sulfur (*closed circles* biomass, *closed triangles* total sulfane in polysulfide and sulfide). **b** Growth with 50 mM formate/2 mM acetate and 10 mM thiosulfate (*closed circles* biomass, *open circles* thiosulfate, *closed triangles* sulfide, *open triangles* sulfite). **c** Growth with 40 mM pyruvate and 30 mM arsenate (*closed circles* biomass, *open circles* arsenite, *open triangles* acetate). **d** Growth with arsenate as *e*-acceptor: influence of arsenate concentration [*closed circles* with 20 mM pyruvate (maximal accumulation of arsenite = 15 mM), *open circles* with 20 mM lactate (maximal accumulation of arsenite = 20 mM), *open triangles* with 50 mM formate (maximal accumulation of arsenite = 30 mM)]. The data in the experiments A and D are average from two independent cultures, the data in the experiments B and C are from a single run

Strain AHT28 is an obligate anaerobe with a respiratory metabolism restricted to the oxidation of a limited range of simple electron donors, such as H_2_, formate, pyruvate and lactate. Growth with hydrogen and formate was possible only in the presence of acetate as a carbon source (i.e. litho-heterotrophy). Among the tested electron acceptors, elemental sulfur, thiosulfate and arsenate can be used for growth with H_2_ and formate, while pyruvate and lactate could only be used with arsenate (Table 1). Growth with excess of elemental sulfur as the *e*-acceptor was accompanied by the accumulation of high concentrations of yellow polysulfide, which is a product of spontaneous reaction of the actual product HS^−^ with the remaining sulfur stable at high pH (Fig. 3a). Reduction of thiosulfate was incomplete with formation of sulfide and sulfite (two-electron reduction, Fig. 3b). The fastest growth was observed with pyruvate as the *e*-donor and arsenate as the *e*-acceptor, whereby pyruvate was incompletely oxidized to acetate and arsenate reduced to arsenite (Fig. 3c). Same final products (acetate and arsenite) were accumulating during growth on lactate and arsenate. In case of pyruvate oxidation to acetate (3 *e*-moles), the theoretical stoichiometry of products is 1 acetate:1.5 arsenite, while it was less than 1 in the AHT28 cultures. In case of lactate, the real stoichiometry (1 acetate:1 arsenite) was also much lower than the theoretical 4*e*-reduction (1 acetate:2 arsenite). One of the possible explanations for this might be an excessive electron usage for anabolism, for example for the synthesis of organic compatible solutes.

**Table 1:** Growth of strain AHT28 by anaerobic respiration at pH 10 and 0.6 M Na^+^

*e*-Donor	*e*-Acceptor	Final products	μ_max_ (h^−1^)
H_2_	Sulfur Thiosulfate Arsenate	Polysulfide Sulfide + sulfite Arsenite	0.032 nd 0.030
Formate	Sulfur Thiosulfate Arsenate	Polysulfide Sulfide + sulfite Arsenite	0.055 nd 0.058
Pyruvate	Arsenate	Acetate + arsenite	0.140
Lactate	Arsenate	Acetate + arsenite	0.078

**Fig. 4:**
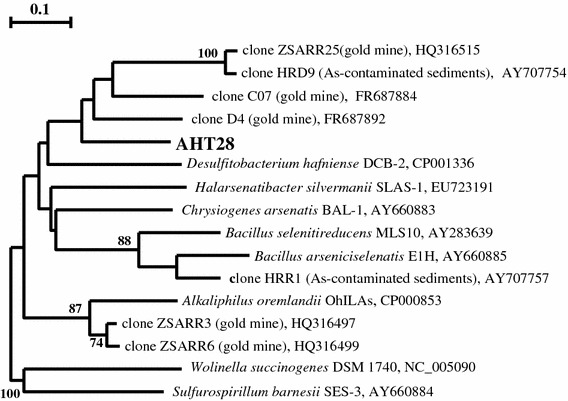
Phylogenetic position of strain AHT28 based on partial translated amino acid sequence of the *aar*A gene. Tree topography and evolutionary distances are obtained by the neighbor-joining method with Jukes and Cantor distances

The organism displayed a high tolerance and activity of conversion of arsenic oxyanions, being able to grow at starting arsenate concentrations up to 40 mM, completely reducing it to arsenite in the presence of excess of *e*-donors, such as H_2_ or pyruvate. The biomass yield during growth with arsenate as *e*-acceptor was proportional to its starting concentration with three different *e*-donors tested indicating that arsenate served as the respiratory electron acceptor (Fig. 3d). This is also corroborated by the presence of the *aar*A gene in the genome of strain AHT28. Despite being a member of the order *Bacillales*, the AHT28 *aar*A is apparently not closely related to the corresponding gene of haloalkaliphilic *Bacillus arseniciselenatis* and *B. selenatireducens* (Fig. 4).

The differential expression of two pathways has also manifested in activity of washed cells pregrown with two different *e*-acceptors. Cells grown with sulfur most actively reduced sulfur and much less actively arsenate with formate as *e*-donor and vice versa with the exception when H_2_ served as *e*-donor. In that case both sulfur-grown and arsenate-grown cells had equally high specific sulfur-reducing activity (Table 2). The latter might indicate a link between sulfur (polysulfide) reductase and hydrogenase in the novel isolate. Finally, cells grown with formate and thiosulfate most actively reduced sulfur, less actively thiosulfate and had practically undetectable arsenate reductase activity (Table 2), which suggests that polysulfide reductase is responsible for both sulfur and thiosulfate reduction in AHT28.

**Table 2:** Comparison of activity of washed cells of strain AHT28 grown with different *e*-acceptors at pH 10 and 0.6 M total Na^+^

*e*-Donor	*e*-Acceptor	Cells grown: formate + sulfur		Cells grown: pyruvate + arsenate		Cells grown: formate + thiosulfate		
		Vs	Va	Vs	Va	Vs	Va	Vt
Formate	Sulfur	50		4		52		
H_2_		40		48		40		
Pyruvate		4		8		nd		
Formate	Arsenate		13		3		1	
H_2_			9		50		0.5	
Pyruvate			2		30		nd	
Formate	Thiosulfate							12
H_2_								9
Pyruvate								0

*Vs* sulfur reduction, *Va* arsenate reduction, *Vt* thiosulfate reduction [in nmol (min mg protein)^−1^], *nd* no data

None of the following electron acceptors can be utilized by strain AHT28 in the presence of formate or lactate as electron donor: nitrate, nitrite, selenate, selenite, ferrihydrite, colloidal MnO_2_, sulfate, sulfite, fumarate. None of the following electron donors in the presence of sulfur and arsenate were able to support growth: acetate, propionate, butyrate, malate, fumarate, succinate, EtOH, PrOH, BuOH, glucose, fructose, xylose, yeast extract.

Arsenate might be present in relatively high concentrations in soda lakes due to mineral feeding from underground waters and evaporative concentration, as, for example, in Mono Lake and Searles Lake, wherein an active microbial dissimilatory arsenic cycle had been demonstrated (Lloyd and Oremland 2006; Kulp et al. 2006, 2007). The dual capacity to grow by dissimilatory respiration of sulfur and arsenate in a haloalkaliphilic organism has only been reported once before for extremely halophilic and alkaliphilic representative of the order *Halanaerobiales*, *Halarsenatibacter silvermanii*, isolated from the hypersaline alkaline Searles Lake (Switzer Blum et al. 2009).

### Influence of pH and sodium on the growth and activity of strain AHT28

**Fig. 5:**
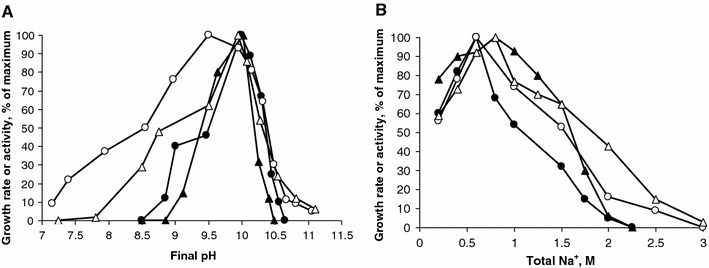
Influence of pH at 0.6 M Na^+^ (**a**) and sodium carbonates at pH 10 (**b**) on anaerobic growth (*closed symbols*) and anaerobic respiratory activity of washed cells (*opened symbols*) of strain AHT28. *Circles* growth or respiratory activity with pyruvate as *e*-donor and arsenate as *e*-acceptor, *triangles* growth or activity with formate as *e*-donor and sulfur as e-acceptor. The data represent mean values obtained in two–three replicates with a deviation ranged from 3 to 12 %

Both with sulfur and arsenate as *e*-acceptors the growth was active at a pH above 8.5 and up to 10.5–10.6, while the activity of washed cells had significantly broader pH range especially toward the lower pH limit (Fig. 5a). It is necessary to stress that in both cases (with arsenate and especially with sulfur) the pH changed significantly during growth and therefore the final pH values are presented in the graphs. In sodium carbonate-based medium at initial pH 10, strain AHT28 was able to grow up to 2 M total Na^+^ with an optimum at 0.6–0.8 M, while the resting cells remained active up to 2.5 M (Fig. 5b). According to these data strain AHT28 can be characterized as moderately salt-tolerant obligate alkaliphile.

Concluding, the *Bacillales* strain AHT28 isolated from sediments of Siberian soda lakes is a highly active haloalkaliphilic anaerobe specialized on respiration of both elemental sulfur and arsenate. Phenotypically, it differs significantly from the known anaerobic representatives of the order *Bacillales* (Table 3) representing a first example with such catabolism within this large bacterial phylum. Together with its distinct phylogenetic position, the overall difference warrants proposal of strain AHT28 as a novel genus and species *Desulfuribacillus alkaliarsenatis* within the order *Bacillales* but with an uncertain family affiliation.

**Table 3:** Phenotypic comparison of strain AHT28 with anaerobic members of the order *Bacillales*

Characteristics	AHT28	*Anaerobacillus arseniciselenatis* ^a^	*Anaerobacillus macyae* ^b^	*Bacillus selenitireducens* ^a,c^
Cell morphology	Curved motile rods, endospores	Nonmotile rods, endospores	Motile rods, endospores	Nonmotile rods
Fermentation	−	Fructose	−	Glucose, fructose, starch
Anaerobic respiration with	Sulfur, thiosulfate, arsenate	Arsenate, selenate, Fe^3+^, fumarate, nitrate	Arsenate, nitrate	O_2_, arsenate, selenite, nitrate, fumarate, TMAO
Electron donors for anaerobic respiration	H_2_, formate, pyruvate, lactate	Lactate, citrate, malate, fructose	H_2_, acetate, pyruvate, succinate, lactate	Lactate, pyruvate, glucose
Catalase	−	+	+	+
DNA G + C mol%	39.1	40.0	37.0	49.0
PH range (optimum)	8.5–10.9 (10.2)	7.0–10.2 (9.8)	Neutrophilic	8.0–10.2 (9.0)
Salt range (M Na^+^)	0.2–2.5	0.2–2.0	0–0.5	0.2–3.7
Habitat	Soda lakes	Soda lakes	Gold mine	Soda lakes

^a^Switzer Blum et al. (1998)

^b^Santini et al. (2004)

^c^ *B. selenitireducens* is a member of the family *Bacillacea*-*1* (see Fig. 2)

## Electronic supplementary material

Below is the link to the electronic supplementary material.

Supplementary Table S1 (PDF 115 kb)

Supplementary material 2 (GB 56 kb)

## Appendix 1

### Description of *Desulfuribacillus* gen. nov.

(De.sul.fu.ri.bacil’lus. L. pref. *de*-, from; L.n. *sulfur*, sulfur; L. masc.n. *bacillus*, a rod; N. L. masc n. *Desulfuribacillus* a sulfur-reducing rod.)

Low GC Gram-positive rod. Obligately anaerobic with respiratory metabolism. Use simple electron donors such as hydrogen and low molecular weight organic acids as electron donors and sulfur and arsenic compounds as electron acceptors. Do not grow autotrophically. Obligately alkaliphilic and moderately salt tolerant. Belongs to the order *Bacillales*. Habitats—soda lakes. The type species is *Desulfuribacillus alkaliarsenatis*.

## Appendix 2

Description of *Desulfuribacillus alkaliarsenatis* sp. nov.

(al.ka.li.ar.sena’tis N.L. n. *alkali* soda ash; N.L. gen. n. *arsenatis*, of arsenate, referring to the ability of the organism to reduce arsenate to arsenite; N.L. adj. *alkaliarsenatis* reducing arsenate at alkaline conditions.)

Cells are rod-shaped, 0.4 × 2–7 μm, motile by several peritrichous flagella. Gram-positive, forming terminal round endospores. Strictly anaerobic, with respiratory catabolism, using elemental sulfur, thiosulfate and arsenate as electron acceptors. Sulfur is reduced to sulfide through polysulfide, thiosulfate is reduced incompletely to sulfide and sulfite and arsenate is reduced to arsenite. Grows lithoheterotrophically with hydrogen and formate in the presence of sulfur, thiosulfate and arsenate and heterotrophically with lactate and pyruvate in presence of arsenate. Obligately alkaliphilic with a pH range for growth between 8.5 and 10.6 and an optimum around 10 and moderately salt tolerant with a range from 0.2 to 2.5 M Na^+^ (optimum at 0.6–0.8 M). Mesophilic, with a maximum temperature for growth at 43 °C and an optimum at 35 °C. The predominant fatty acids in the polar membrane lipids include 16:0, 16:1ω7c and 18:1ω7c. The dominant respiratory lipoquinone is MK-7. meso-DAP is present in the murein. The G + C content of the genomic DNA is 39.1 mol% (T_m_). The type strain is AHT28^T^ (DSM24608^T^ = UNIQEM U855^T^). Isolated from sediments of soda lakes in southwestern Siberia. The GenBank 16S rRNA gene sequence accession number of the type strain is HM046584.
