# Supplementary material for: Desulfuribacillus alkaliarsenatis gen. nov. sp. nov., a deep-lineage, obligately anaerobic, dissimilatory sulfur and arsenate-reducing, haloalkaliphilic representative of the order Bacillales from soda lakes
Source: Extremophiles. 2012 May 24;16(4):597–605. doi: 10.1007/s00792-012-0459-7 (PMC3386488; doi:10.1007/s00792-012-0459-7)
Supplement: Supplementary file 1 — Supplementary Table S1 (PDF 115 kb) [file 792_2012_459_MOESM1_ESM.pdf]

Supplementary table S1. Basic parameters of soda lakes in Kulunda Steppe (Altai, Russia) in July 2010 used for isolation of strain AHT28

A: Brine characteristics

Lake	pH	Salinity, g/l	Alkalinity, M		Appearance
			Na_2CO_3	Total	Brine
Cock Soda	10.10	70	0.34	0.68	clear
Bitter-1	10.15	400	2.00	4.40	oily yellow
Bitter-2	10.25	80	0.50	1.00	clear
Tanatar-1	9.90	400	1.20	2.40	oily yellow
Tanatar-3	10.03	110	0.50	1.00	clear
Tanatar-5	9.90	170	0.80	1.90	muddy

B: Sediments

Lake	Sediment layer	Acid labile sulfides (HS^-/FeS)
Cock Soda lake	10-20	**5.81**
	21-30	**6.60**
	31-40	**6.07**
Bitter-1	10-15	0.05
	16-20	1.91
	21-25	**17.44**
Tanatar-5	15-20	0.87
	21-25	0.94
	26-30	1.65

Supplementary Table S1

PLFA profiles of strain AHT28 in comparison with the anaerobic alkaliphilic representatives of the order *Bacillales*. Bacteria were grown at pH 10, 30°C up to the late logarithmic phase. Only FA above 0.5% are shown. Major compounds are highlighted. 1 – our data; 2 – Zavarzina et al. 2009

FA	**AHT28**	*Natronobacillus diazotrophicus*[1]	*Anaerobacillus alkalidiazotrophicus*[1]	*Anaerobacillus alkalilacustris*[2]
i13:0		**6.3**		
ai13:0		**7.6**		
i14:0		1.2		
14:0	0.7	3.6	**10.2**	
14:1 ω5			1.2	
15:0		3.2		
i15	0.5	**14.3**	2.9	
15:1ω4				
15:1ω6				
i15:1			1.5	
ai15:0		**46.4**	**12.6**	**12.0**
ai15:1			1.7	
16:0	**24.6**	**7.5**	**30.7**	**31.1**
16:1ω5	3.5			
16:1ω5a				
16:1ω7				
16:1ω7a	1.3		**29.0**	
16:1ω7c	**20.0**			**23.2**
16:1ω9	**6.6**			
16:1ω9a	2.3			
16:1ω9c				
16:0a	2.8			
i16:0		1.2	0.9	
i16:1ω7				
i16:1ω5a				
16:1ω5 DMA				
16:1ω7 DMA				
i17	1.1	1.7	0.5	
ai17:0		**7.2**	1.2	
17:1	0.8			
i17:1			0.7	
18:0	1.4		2.4	
18:1ω5	1.4		0.5	
18:1ω6				
18:1ω6a				
18:1ω7				
18:1ω7a	4.1			
18:1ω7c	**20.7**		3.8	**8.4**
18:1ω9	6.0		1.2	
18:1ω9a	1.0			
i18:1ω6a				
i18:1ω7a				
18:1ω7DMA			1.4	
18:2				
